# Pitfalls of Computed Tomography in the Coronavirus 2019 (COVID-19) Era: A New Perspective on Ground-Glass Opacities

**DOI:** 10.7759/cureus.8151

**Published:** 2020-05-16

**Authors:** Sara Mehrabi, Silvia Fontana, Francesca Mambrin, Hoang Quyen Nguyen, Elda Righi, Evelina Tacconelli, Giancarlo Mansueto

**Affiliations:** 1 Radiology, Azienda Ospedaliera Universitaria Integrata Verona, Verona, ITA; 2 Infectious Diseases, Azienda Ospedaliera Universitaria Integrata Verona, Verona, ITA

**Keywords:** covid 19, ground-glass opacity, ct, computed tomography, emphysema, interstitial pneumonia, multi-viral pneumonia, lung adenocarcinoma

## Abstract

Aim

To study ground-glass opacities (GGO) not only from the coronavirus 2019 (COVID-19) pneumonia” perspective but also as a radiological presentation of other pathologies with comparable features.

Methods

We enrolled 33 patients admitted to Policlinico Universitario G. B. Rossi who underwent non-contrast-enhanced (NCE) or contrast-enhanced (CE) chest computed tomography (CT) between March 12 and April 12. All patients with CT-detected ground-glass opacity (GGO) were included. All patients resulted as COVID-19 negative at the reverse transcription-polymerase chain reaction (RT-PCR) assay. We studied the different pathologies underlying GGO features: neoplastic diseases and non-neoplastic diseases (viral pneumonias, interstitial pneumonias, and cardiopulmonary diseases) in order to avoid pitfalls and to reach the correct diagnosis.

Results

All CT scans detected GGOs. Symptomatic patients were 25/33 (75.7%). At the clinical presentation, they reported fever and dry cough; in six out of 25 cases, dyspnea was also reported (24%). Thirty-three (33; 100%) showed GGO at CT: 15/33 (45.45%) presented pure GGO, and 18/33 (54.54%) showed GGO with consolidation. The RT-PCR assay was negative in 100%. We investigated other potential underlying diseases to explain imaging features: neoplastic causes (8/33, 24.24%) and non-neoplastic causes, in particular, infectious pneumonias (16/33, 48,48 %, viral and fungal), interstitial pneumonias (4/33, 12,12%), and cardio-pulmonary disease (5/33, 15,15%).

Conclusions

GGO remains a diagnostic challenge. Although CT represents a fundamental diagnostic tool because of its sensitivity, it still needs to be integrated with clinical data to achieve the best clinical management. In the presence of typical imaging features (e.g. GGO and consolidation), the radiologist should focus on the pandemic and manage a suspect patient as COVID-19 positive until proven to be negative.

## Introduction

Coronavirus 2019 (COVID-19) pneumonia is caused by a novel virus from the Coronaviridae family, from which the severe acute respiratory syndrome (SARS) and Middle East respiratory syndrome (MERS) viruses also originated, causing two epidemics in 2002 and 2012, respectively. According to the previous study, the disease develops in three phases: early (Days 1-3), intermediate (Days 4-6), late (after Day 6) [1]. Even if chest CT can detect lung anomalies before symptom onset, the findings follow a temporal pattern and are consistent with clinicopathological development: the first and most common feature is ground-glass opacity (GGO), which represents early alveolar damage, with bilateral subpleural distribution in the lower lobes; then, areas of consolidation appear and tend to coalesce on the underlying GGOs. Later, in this background, the “crazy paving” pattern can be seen, with thickened interlobular septa and intralobular lines. Lymphadenopathies and pleural effusion are rarely seen [2].

Chest CT plays a pivotal role in the detection and early management of COVID-19 pneumonia; despite its sensitivity, it lacks specificity because of the wide spectrum of pathologies that can show GGO. The aim of our study is to show chest CT findings that could mimic COVID-19 pneumonia; in the COVID-19 era, we should not forget other lung diseases that could be misinterpreted in our experience. It is, therefore, fundamental to correlate CT findings with clinical history and lab data to achieve the correct diagnosis and an integrated scenario for every patient [3].

Ground-glass opacity is deﬁned as hazy opacity that does not obscure underlying bronchial structures or pulmonary vessels at computed tomography (CT). It represents many histopathological processes because the underlying mechanism may be partial airspace ﬁlling; interstitial thickening with inﬂammation, edema, ﬁbrosis; and neoplastic proliferation. GGO is a frequent focal lung finding representing cancer; it can also represent lung infections, lung edema, or interstitial diseases (where GGO can represent disease activity and may precede irreversible fibrosis). Since it is a non-specific radiological finding, it is fundamental to know the underlying physiopathology to narrow the differential diagnosis [4].

## Materials and methods

We retrospectively enrolled 33 patients aged between 19 and 88 years (20 males and 13 females), admitted to Verona A.O.U.I. - Policlinico Universitario G. B. Rossi, who underwent non-contrast-enhanced (NCE) and contrast-enhanced (CE) chest CT between March 12 and April 12. When CE-CT was performed, we used a high iodine concentration contrast agent (Ultravist 370, Bayer Schering Pharma AG, Berlin, Germany). We included all patients with CT images positive for GGO. COVID-19 patients were excluded from this study. Patients tested for COVID-19 underwent a reverse transcription-polymerase chain reaction RT-PCR assay on a nasopharyngeal swab. Patients with other infective pneumonia were confirmed with bronchoalveolar lavage (BAL), viral panel, serologic tests, or blood cultures.

CTs were performed with the patient supine at full inspiration, using a 64-row MDCT scanner (Brilliance iCT, Philips Healthcare, Amsterdam, Netherlands). Scanning parameters were tube voltage 120 kV and tube current modulation 100 mA. Images were reconstructed with a slice thickness of 1 mm or 2 mm. All images were reconstructed on both lung (width, 1500 HU; level: -700 HU) and mediastinal (width, 350 HU; level: 40 HU) settings.

## Results

The number of symptomatic patients was 25/33 (75.7%). At clinical presentation, they reported fever and dry cough, and six out of 25 (24%) cases also reported dyspnea. Thirty-three (33; 100%) showed GGO at CT; 15/33 (45.45%) presented pure GGO and 18/33 (54.54%) presented GGO with consolidation. COVID-19 pneumonia was suspected because of the CT report and clinical presentation so an RT-PCR assay by nasopharyngeal swab was performed; interestingly, the virus was not detected in any patient. We investigated other potential underlying diseases: neoplastic causes (8/33, 24.24%) and non-neoplastic causes, in particular, infectious pneumonias (16/33, 48,48 %, viral and fungal), interstitial pneumonias (4/33, 12,12%), and cardio-pulmonary disease (5/33, 15,15%). The different meaning of GGO in every category was also investigated.

## Discussion

Since GGO lesions can be caused by a wide spectrum of pathologies, a GGO is a non-specific radiological finding. It does not represent a confirmed diagnosis, even in the Covid-19 era. It is, therefore, necessary to understand the underlying histopathology and to integrate imaging with clinical and lab data to achieve the correct diagnosis. According to our experience, in the current paper, we distinguished between the neoplastic and non-neoplastic causes of GGO. For each category, we analyzed the diagnostic pitfalls and discussed management during radiological practice.

Lung adenocarcinoma

**Figure 1:**
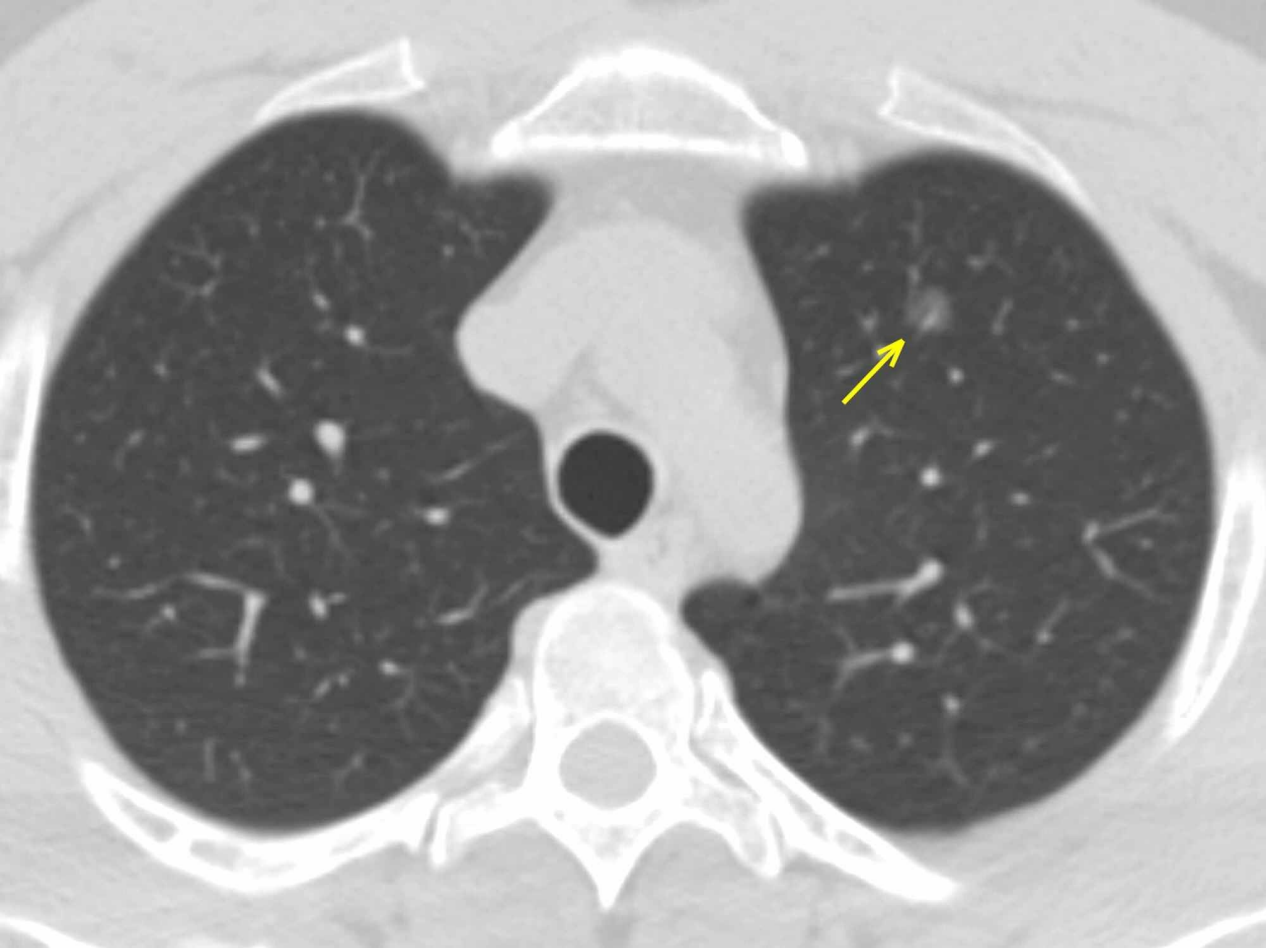
Carcinoma in situ - axial section A single ground-glass lesion in the apical segment of the superior left lobe (arrow). The localization allowed ruling out the possibility of COVID-19 pneumonia. COVID-19: coronavirus 2019

**Figure 2:**
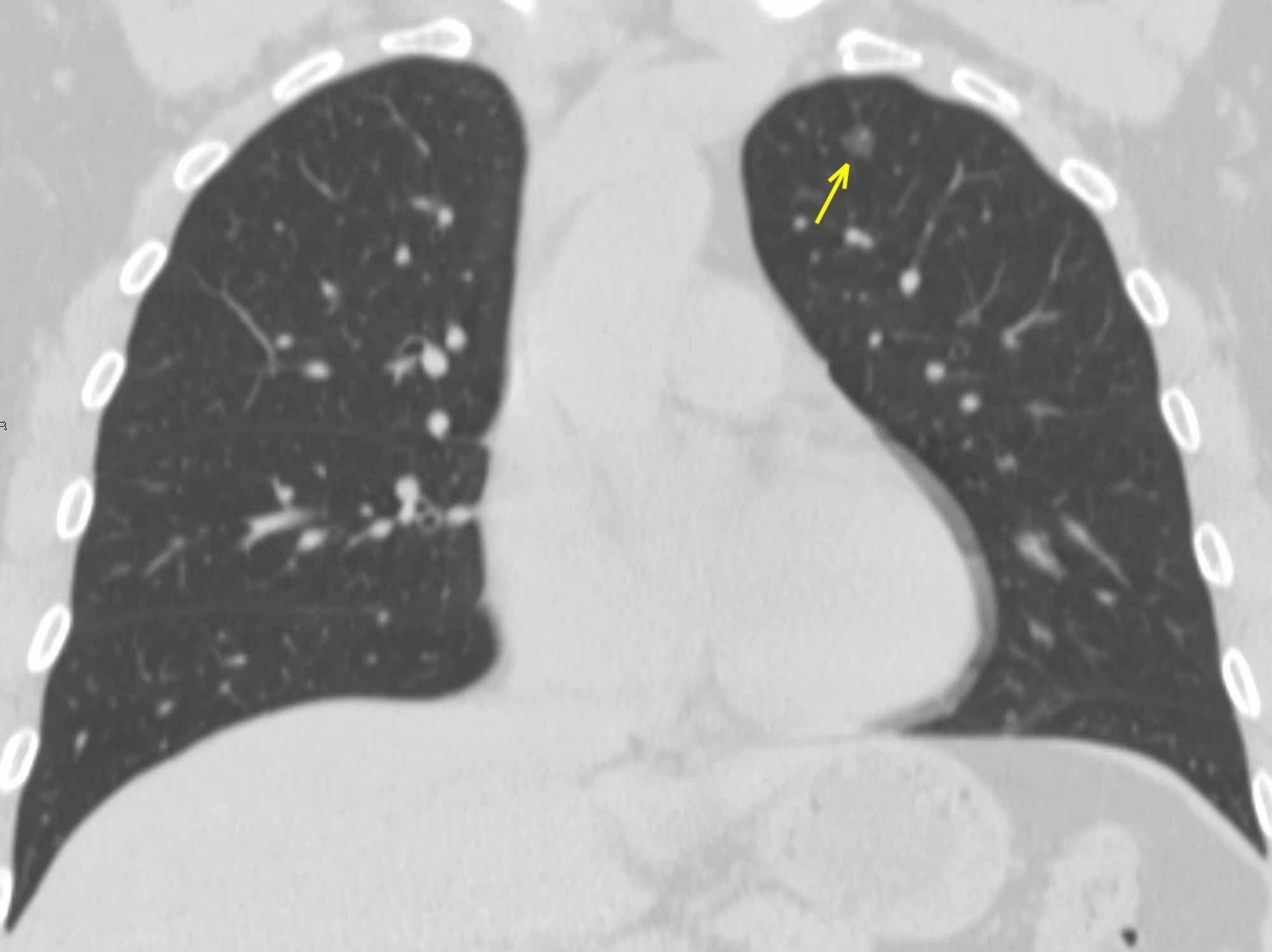
A carcinoma in situ - coronal section A single ground-glass lesion in the apical segment of the superior left lobe (arrow). The localization allowed ruling out the possibility of COVID-19 pneumonia. COVID-19: coronavirus 2019

A 39-year-old male patient, a health worker, presented to the emergency department, with a history of dry cough. He did not report any significant comorbidity, nor malignancies in his clinical history. In suspicion of COVID-19 pneumonia (clinical and epidemiological criteria), an RT-PCR assay on nasopharyngeal swab was performed. Later, he underwent a chest CT scan. The report showed a pure ground-glass nodule in the apical segment of the left superior lobe (Figures 1-2). The result of the RT-PCR was negative.

Considering the mild symptomatology and the presence of pure GGO, early-stage COVID-19 pneumonia could have been diagnosed; nevertheless, it is important to consider lung cancer as an incidental finding. According to the Fleischner Society Guidelines, a 12-months follow-up was recommended [5].

GGO is a common sign of lung cancer. According to the World Health Organization (WHO) classification, adenocarcinoma and its precursors are classified into preinvasive lesions (atypical adenomatous hyperplasia (AAH) and adenocarcinoma in situ (AIS)), minimally invasive adenocarcinoma (MIA), typically presenting as a pure GGO nodule greater than 10 mm in diameter, and invasive adenocarcinoma. In general, lung adenocarcinomas are thought to follow a path in which AAH progresses to AIS, followed by invasive adenocarcinoma. Most pure ground-glass nodules (GGN - without solid component) are preinvasive adenocarcinomas, often behaving as indolent tumors; semisolid nodules tend to be adenocarcinomas, as well as invasive because of the intrinsic histological difference in the solid component: the more they grow, the more invasive they are [6].

The GGOs of AIS usually manifest as regions of slightly higher attenuation relative to the opacity of AAH due to the histopathologic difference in the number of cellular components within the nodule or the thickness of alveolar walls. Pure GGNs greater than 16.4 mm in diameter have been reported to represent invasive adenocarcinoma [7-8].

Infectious disease: viral pneumonia

**Figure 3:**
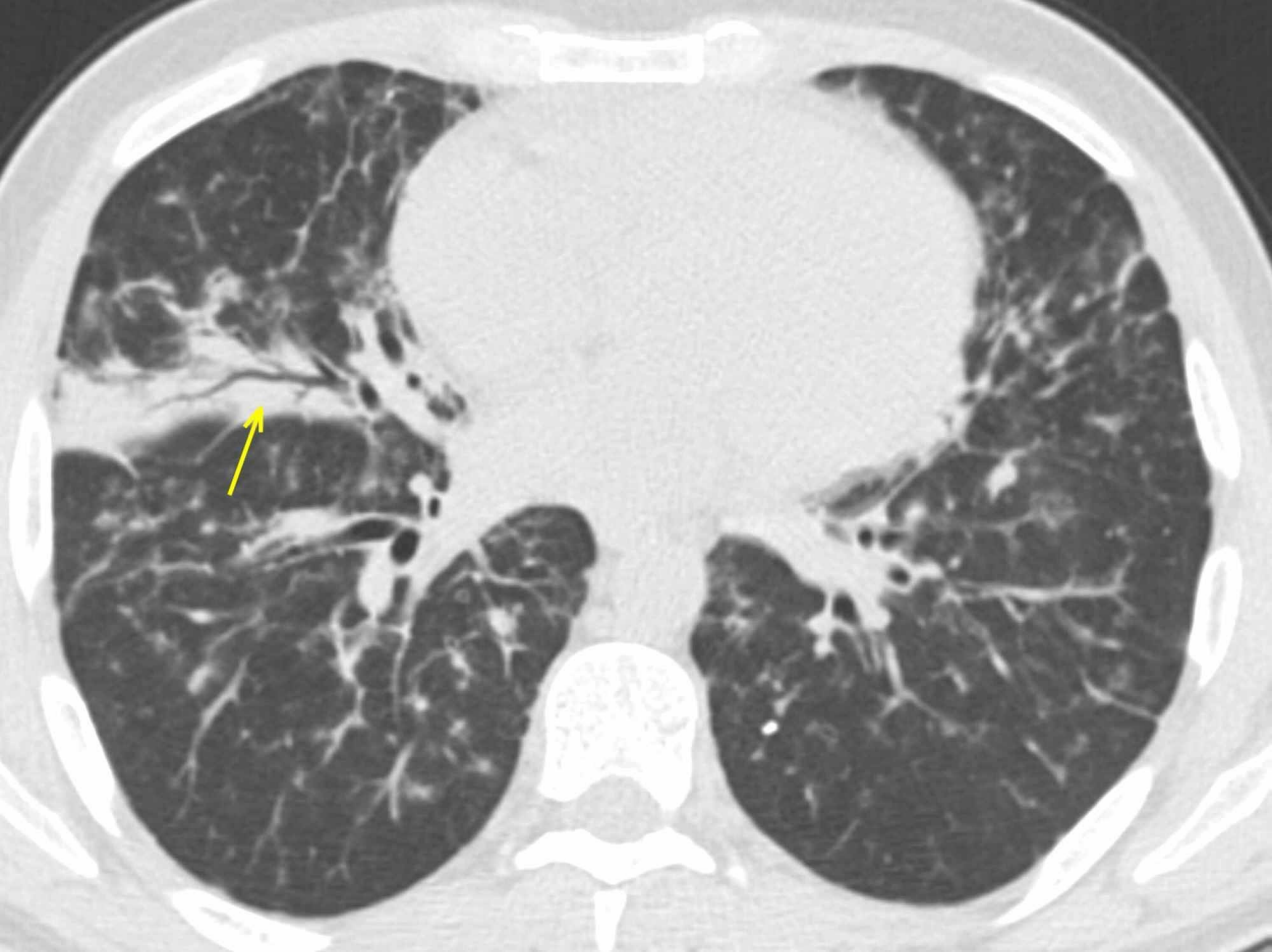
Influenza A bilateral pneumonia Bilateral consolidating areas in the inferior right lobe with air bronchogram (arrow); the absence of a pure GGO, along with clinical and lab data, allowed the exclusion of COVID-19 pneumonia. GGO: ground-glass opacity; COVID-19: coronavirus 2019

**Figure 4:**
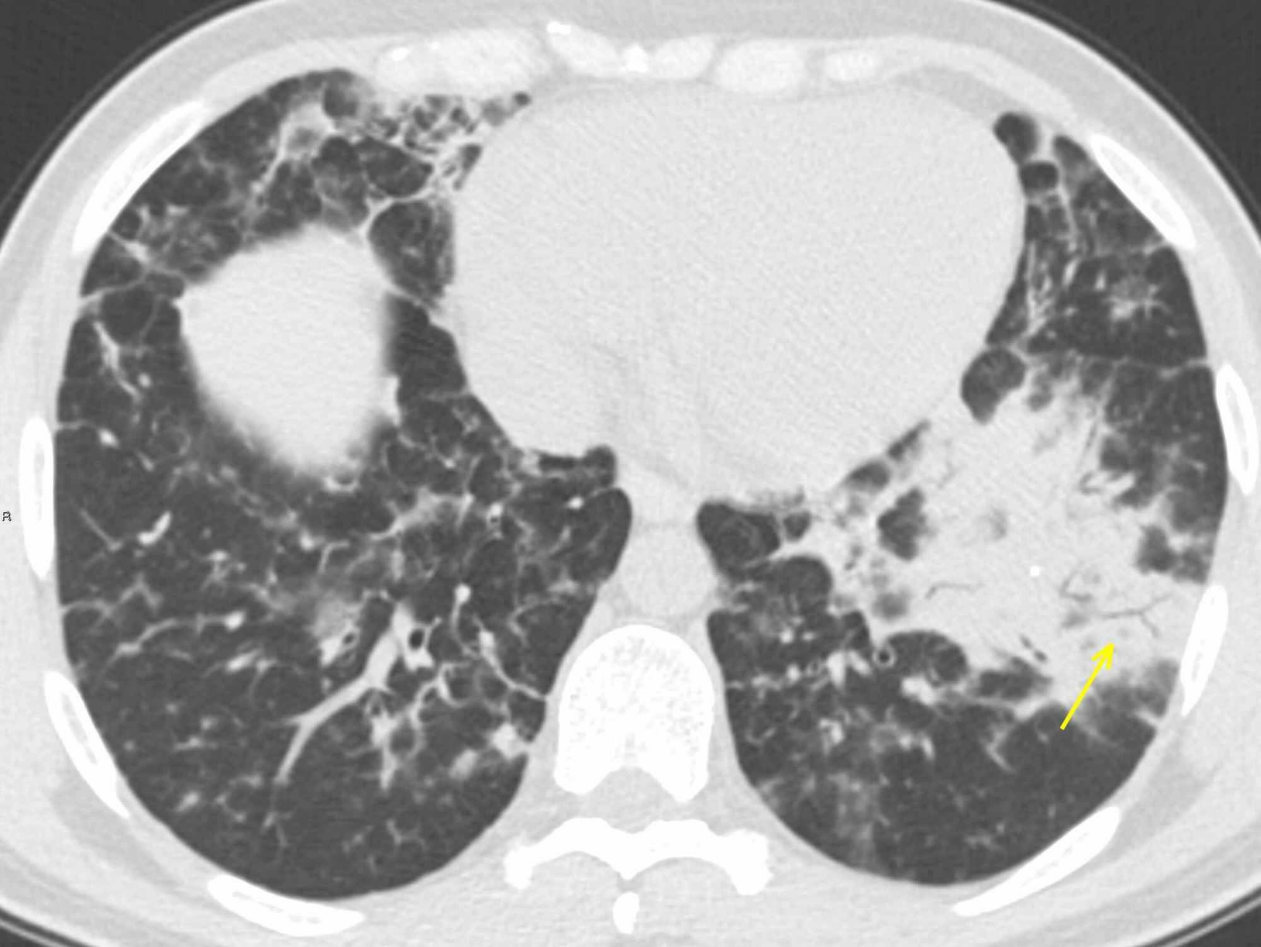
Influenza A bilateral pneumonia Bilateral consolidating areas in the inferior left lobe with air bronchogram (arrow); the absence of pure GGO along with clinical and lab data allowed the exclusion of COVID-19 pneumonia. GGO: ground-glass opacity; COVID-19: coronavirus 2019

A 32-year-old male presented with a two-week history of high fever (T-max 39.5°C), cough, and muscle ache. Blood tests showed mild lymphopenia (900/mmc) and high C-reactive protein (CRP) levels (56 mg/L, normal value <5); therefore, an ongoing infective process was suspected. Considering the possibility of COVID-19 pneumonia, RT-PCR was performed twice for the persistence of typical symptoms and because of chest CT findings, highly suspect for viral pneumonia. The CT scan reported a consolidating area in the medial segment of the middle lobe and GGOs in the lower left lobe (Figures 3-4). Since the RT-PCR persisted negative, a BAL was performed and influenza A virus detected by multiplex PCR. The differential diagnosis among viral pneumonias appears to be extremely difficult: there is a significant overlap in the imaging appearance and overlapping with other inflammatory lung diseases; furthermore, blood exams are highly unspecific, too, showing generally lymphopenia and slightly increased CRP.

The CT patterns of viral pneumonia are related to the pathogenesis of the viral infection and are affected by the immune status of the host and the underlying pathophysiology of the viral pathogen. Moreover, viruses of the same family have similar pathogenesis; consequently, viral pneumonia caused by different viruses from the same virus family exhibits a similar pattern on chest CT images [9-10]. GGOs represent areas of alveolar damage and, in patients developing acute respiratory distress syndrome (ARDS), fluid-refilled alveoli. COVID-19 is a new type of viral pneumonia; previous studies have demonstrated frequent CT findings, in particular, bilateral GGO and interstitial thickening, similar to other viral pneumonias, specifically SARS, and MERS, belonging to the same Viridae. SARS and MERS outbreaks were also due to a coronavirus: consequently, studying and understanding those epidemics may be helpful in managing the current pandemics [11].

A and B influenza viruses belong to the Orthomyxoviridae family. In individuals with chronic comorbidities (diabetes, cardiac failure), severe complications leading to ARDS may occur.

Chest CT shows bilateral reticular areas of opacity, sometimes with focal areas of consolidation, usually in the lower lobes. Patchy GGOs can be associated with the areas of consolidation. Pleural effusion is rare.

Interstitial pneumonias

**Figure 5:**
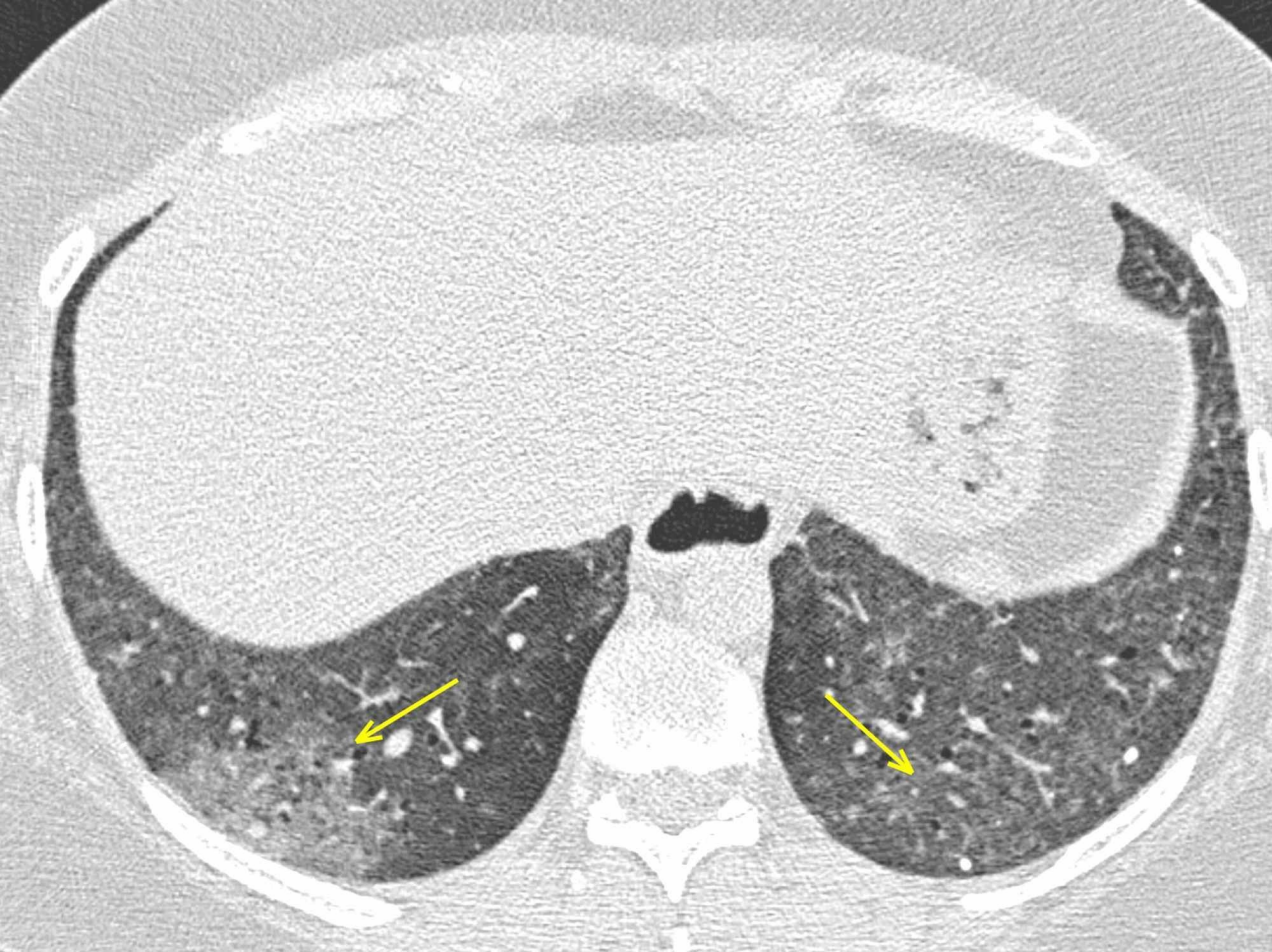
LIP in Sjogren syndrome - axial section The female patient affected by Sjogren syndrome with LIP. Patchy GGOs (arrows) seen predominantly in the lower lobes. The patient developed fever and dyspnea. Despite the typical onset symptoms and the presence of imaging features suspect for COVID-19, RT-PCR was negative. LIP: lymphocytic interstitial pneumonia; GGO: ground-glass opacity; COVID-19: coronavirus 2019; RT-PCR: reverse transcription-polymerase chain reaction

**Figure 6:**
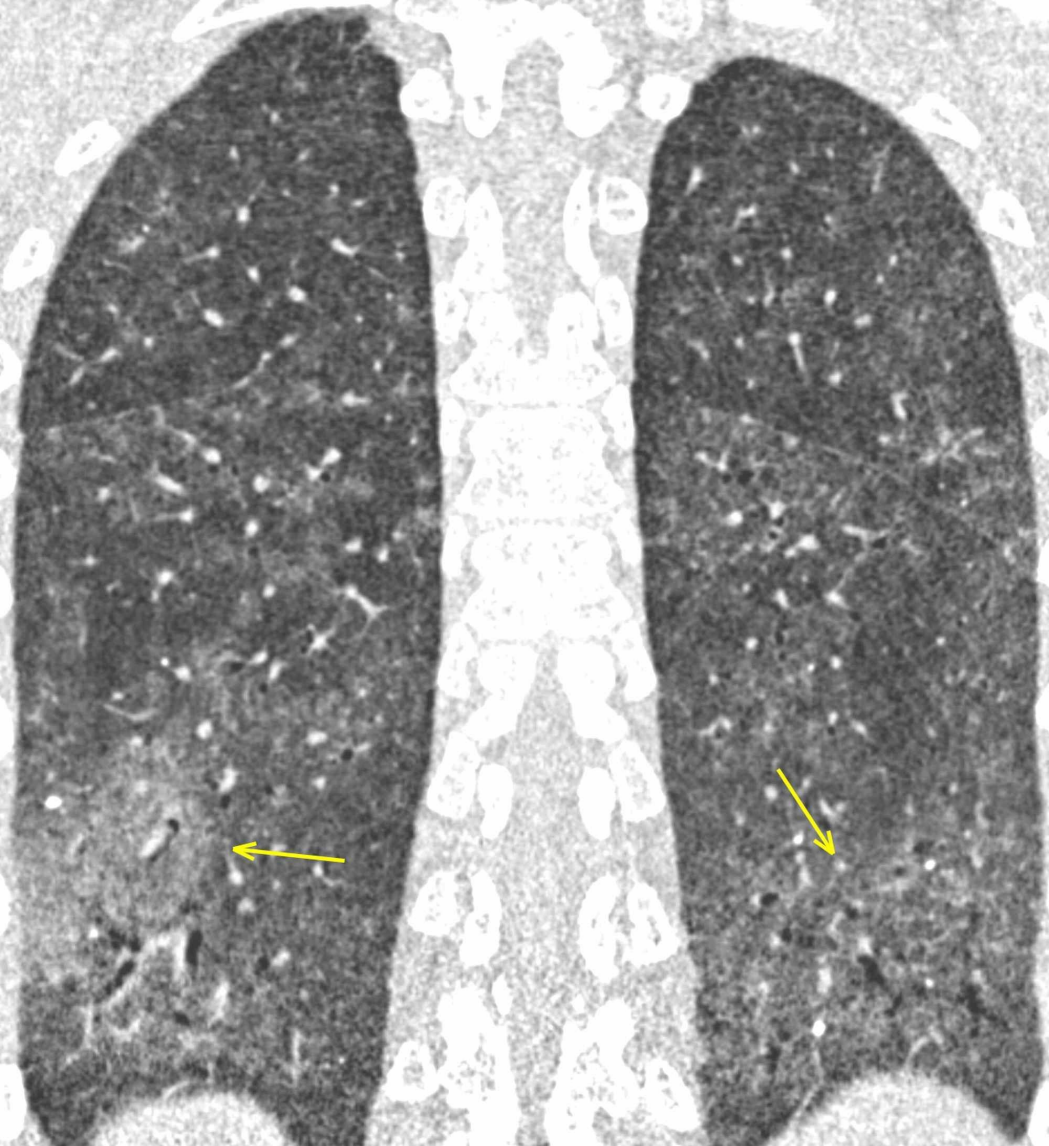
LIP in Sjogren syndrome - coronal section The female patient affected by Sjogren syndrome with LIP. Patchy GGOs (arrows) seen predominantly in the lower lobes. The patient developed fever and dyspnea. Despite the typical onset symptoms and the presence of imaging features suspect for COVID-19, RT-PCR was negative. LIP: lymphocytic interstitial pneumonia; GGO: ground-glass opacity; COVID-19: coronavirus 2019; RT-PCR: reverse transcription-polymerase chain reaction

A 39-year-old female affected by Sjögren syndrome underwent a scheduled chest CT scan to assess lung involvement. GGOs were reported bilaterally, predominantly in the lower lobes, along with interstitial thickening (Figures 5-6). Despite the typical imaging features and the onset of mild symptoms, the report excluded COVID-19 pneumonia; interstitial inflammatory involvement was correctly diagnosed. CT findings, along with clinical history, led to the correct diagnosis: it could have been misinterpreted since, in the COVID-19 era, every patient should be considered positive until proven negative. RT-PCR confirmed negative.

Interstitial pneumonias are a heterogeneous group of diffuse parenchymal lung diseases caused by a combination of inflammation and fibrosis. The primary site of injury is the interstitium, which includes the space between the epithelial and endothelial membranes; airspaces, airways, and vessels are often affected [12]. Interstitial pneumonias may be idiopathic or secondary to a variety of other causes, including collagen vascular diseases, pneumoconiosis, infection, and smoking. The American Thoracic Society and European Respiratory Society classification includes seven clinicopathologic entities: idiopathic pulmonary fibrosis (IPF), nonspecific interstitial pneumonia (NSIP), cryptogenic organizing pneumonia, acute interstitial pneumonia (AIP), respiratory bronchiolitis (RB)-associated interstitial lung disease (ILD), desquamative interstitial pneumonia (DIP), and lymphoid interstitial pneumonia (LIP) [13].

LIP is regarded as a variant of diffuse pulmonary lymphoid hyperplasia primarily affecting the interstitium and it is distinguished from low-grade malignant lymphoproliferative diseases with immunohistochemical analysis. LIP is frequently associated with systemic disorders (Sjögren syndrome, acquired immunodeficiency virus (AIDS), Hashimoto thyroiditis) [14].

Abnormalities on CT in patients with LIP are usually bilateral and may be diffuse or have lower lung predominance. A typical finding is GGO, representing diffuse interstitial phlogistic involvement, along with interlobular thickening. Centrilobular nodules and perivascular cysts are also common, representing inflammatory infiltration of the peribronchiolar interstitium [15].

Chronic obstructive pulmonary disease (COPD): emphysema

**Figure 7:**
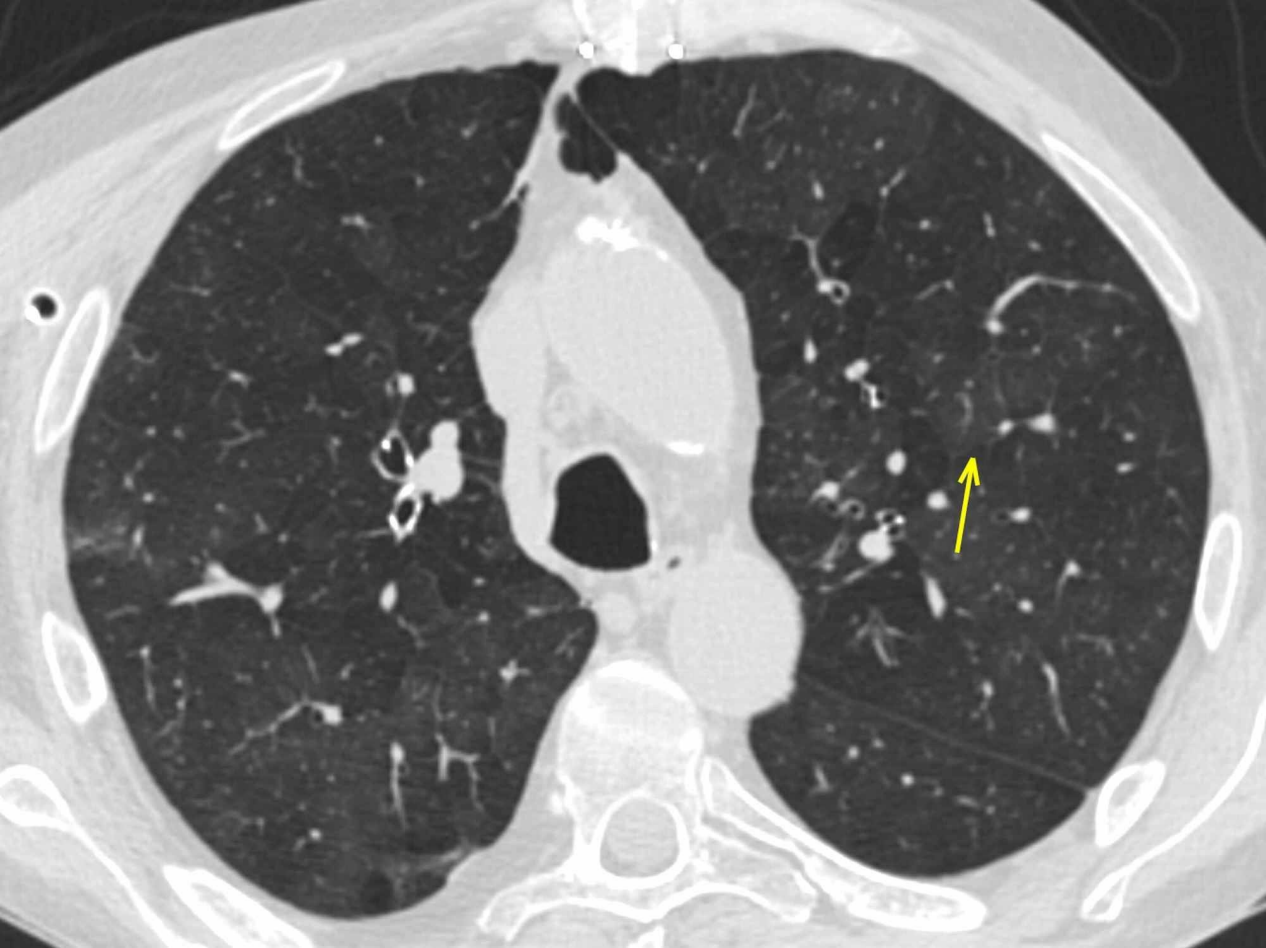
Emphysema Patchy GGOs (arrow) in a patient with a cough who reported a COVID-19-positive contact. GGO: ground-glass opacity; COVID-19: coronavirus 2019

A 54-year-old male underwent a heart transplant due to severe dilatative cardiomyopathy associated with mitral failure. He also reported centrilobular emphysema in his clinical history. During hospitalization and rehabilitation, direct contact with a COVID-19-positive patient was reported. Since the patient developed a cough, a chest CT scan was performed, reporting patchy bilateral ground-glass opacities affecting both the superior and inferior lobes, along with mild bronchiectasis (Figure 7).

**Figure 8:**
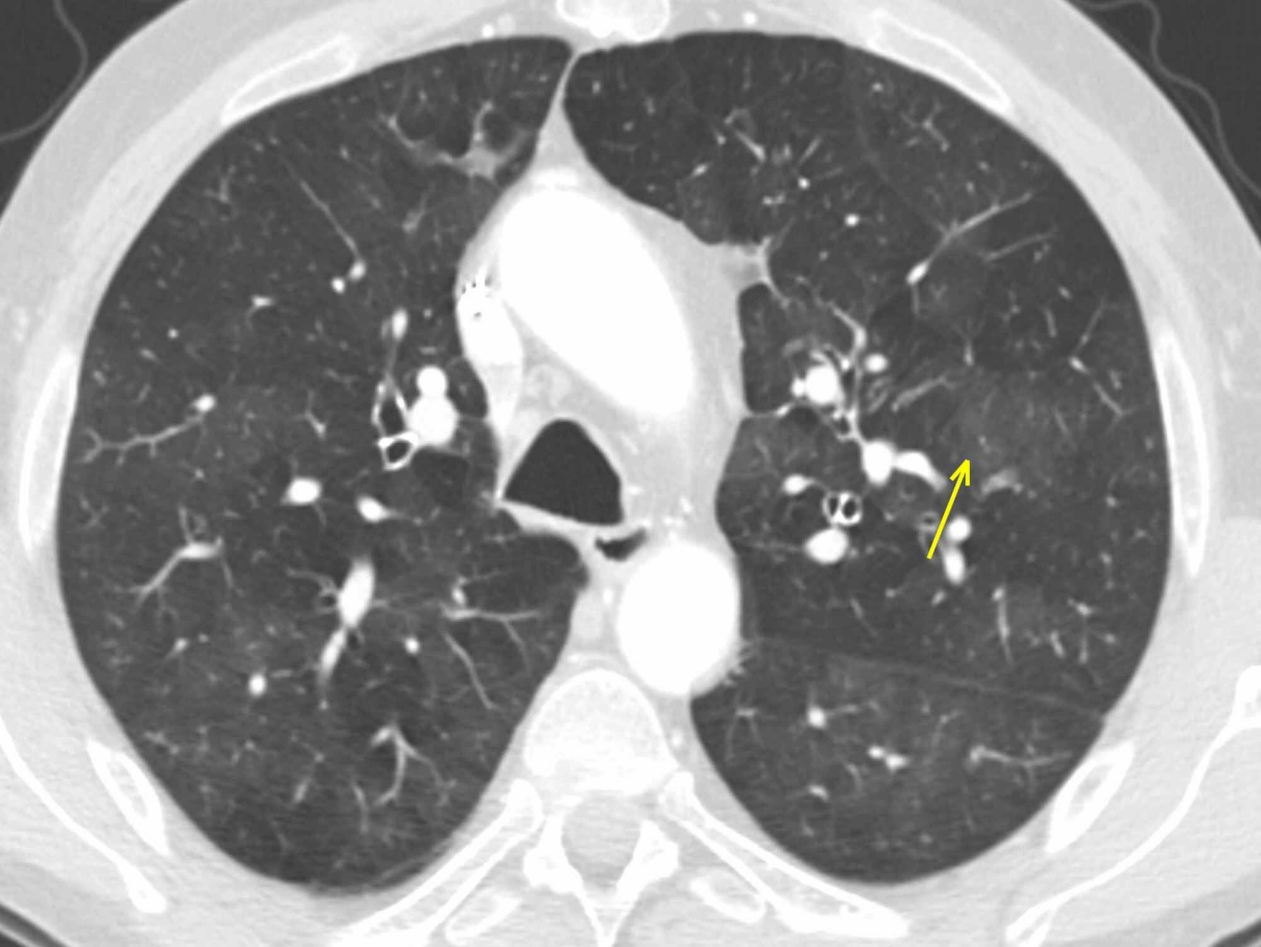
Emphysema The same patient as in Figure 7: a careful review of the CT performed in January 2019 shows identical bilateral GGOs (arrow), confirming imaging features as consistent with emphysema and, therefore, ruling out COVID-19 pneumonia. CT: computed tomography; GGO: ground-glass opacity; COVID-19: coronavirus 2019

A careful review of the previous CT showed diffuse ground-glass opacities bilaterally, completely similar to the most recent lung findings: COVID-19 pneumonia was excluded (Figure 8). The RT-PCR negative swab confirmed the radiological report.

CT in centrilobular-emphysema shows small, well-defined or poorly defined areas of low attenuation surrounded by normal lung; centrilobular pulmonary arteries or arterioles mark the center of each lobule. The low-attenuation areas may range from 1-3 mm. Small airway disease is often a component of both emphysema-predominant disease and airway-predominant disease involving larger airways. Isolated small airway disease can also occur as a primary expression of COPD [16].

In addition to centrilobular nodules, CT in cigarette smokers could show the typical imaging features of infiltrative lung disease, including GGOs and reticular anomalies, which likely correspond to variable combinations of respiratory bronchiolitis, airspace enlargement with fibrosis, and smoking-related interstitial fibrosis [17]. In these findings, the histopathological mechanism underlying GGO is small airway obstruction or fibrosis, which causes vasoconstriction in that area of the lung, shunting blood away from areas of impaired gas exchange, with hyperperfusion of the adjacent normal lung, leading to relatively increased attenuation with a subsequent “mosaic attenuation” aspect [18-19].

GGO seen on CT can represent a wide spectrum of pathologic conditions, such as inflammatory disease and lung neoplasms. The analysis of several parameters, such as bilateral/unilateral involvement localization in the lung parenchyma, superimposed consolidation, and the presence of lymphadenopathies and pleural effusion and associated abnormalities will avoid most pitfalls, indicating a possible diagnosis of the underlying cause and offering a consistent differential diagnosis. Nevertheless, GGO remains a diagnostic challenge and, therefore, a systematic approach is necessary to ensure an optimal workup.

## Conclusions

COVID-19 pneumonia is a novel disease, therefore, special attention to differential diagnoses with clinical and radiological presentations that may mimic it is recommended. Although CT represents a fundamental diagnostic tool because of its sensitivity, it still needs to be integrated with clinical data to achieve the best clinical management. This principle should be applied not just in the COVID-19 era but also in all pathologies that could have very similar radiological findings. However, in the presence of typical imaging features (e.g. GGO and consolidation), the radiologist should focus his attention on the pandemic and manage a suspect patient as COVID-19 positive until proven to be negative since it is of primary importance to manage COVID-19 pneumonia as early as possible in order to prevent the contagion from continuing to spread.

## References

[1] (2020). World Health Organization. Novel coronavirus - China. https://www.who.int/csr/don/12-january-2020-novel-coronavirus-china/en/.

[2] Zhu N, Zhang D, Wang W (2020). A novel coronavirus from patients with pneumonia in China. N Engl J Med.

[3] Pan Y, Guan H, Zhou S (2020). Initial CT findings and temporal changes in patients with the novel coronavirus pneumonia (2019-nCoV): a study of 63 patients in Wuhan, China. Eur Radiol.

[4] Kligerman SJ, Henry T, Lin CT, Franks TJ, Galvin JR (2015). Mosaic attenuation: etiology, methods of differentiation, and pitfalls. Radiographics.

[5] Hansell DM, Bankier AA, MacMahon H, McLoud TC, Müller NL, Remy J (2008). Fleischner Society: glossary of terms for thoracic imaging. Radiology.

[6] Yanagawa M, Tanaka Y, Kusumoto M (2010). Automated assessment of malignant degree of small peripheral adenocarcinomas using volumetric CT data: correlation with pathologic prognostic factors. Lung Cancer.

[7] Lee HY, Choi YL, Lee KS, Han J, Zo JI, Shim YM, Moon JW (2014). Pure ground-glass opacity neoplastic lung nodules: histopathology, imaging, and management. AJR Am J Roentgenol.

[8] Pedersen JH, Saghir Z, Wille MM, Thomsen LH, Skov BG, Ashraf H (2016). Ground-glass opacity lung nodules in the era of lung cancer CT screening: radiology, pathology, and clinical management. Oncology.

[9] Kim EA, Lee KS, Primack SL (2002). Viral pneumonias in adults: radiologic and pathologic findings. Radiographics.

[10] Miller WT Jr, Mickus TJ, Barbosa E Jr, Mullin C, Van Deerlin VM, Shiley KT (2011). CT of viral lower respiratory tract infections in adults: comparison among viral organisms and between viral and bacterial infections. AJR Am J Roentgenol.

[11] Shi H, Han X, Jiang N (2020). Radiological findings from 81 patients with COVID-19 pneumonia in Wuhan, China: a descriptive study. Lancet Infect Dis.

[12] Mueller-Mang C, Grosse C, Schmid K, Stiebellehner L, Bankier AA (2007). What every radiologist should know about idiopathic interstitial pneumonias. Radiographics.

[13] American Thoracic Society, European Respiratory Society (2002). American Thoracic Society/European Respiratory Society International multidisciplinary consensus classification of the idiopathic interstitial pneumonias. Am J Respir Crit Care Med.

[14] Swigris JJ, Berry GJ, Raffin TA, Kuschner WG (2002). Lymphoid interstitial pneumonia. A narrative review. Chest.

[15] Silva CI, Flint JD, Levy RD, Müller NL (2006). Diffuse lung cysts in lymphoid interstitial pneumonia: high-resolution CT and pathologic findings. J Thorac Imaging.

[16] Lynch DA, Austin JH, Hogg JC (2015). CT-definable subtypes of chronic obstructive pulmonary disease: a statement of the Fleischner Society. Radiology.

[17] Katzenstein AL, Mukhopadhyay S, Zanardi C, Dexter E (2010). Clinically occult interstitial fibrosis in smokers: classification and significance of a surprisingly common finding in lobectomy specimens. Hum Pathol.

[18] Kligerman SJ, Henry T, Lin CT, Franks TJ, Galvin JR (2015). Mosaic attenuation: etiology, methods of differentiation, and pitfalls. Radiographics.

[19] Heyneman LE, Ward S, Lynch DA, RemyJardin M, Johkoh T, Müller NL (1999). Respiratory bronchiolitis, respiratory bronchiolitis-associated interstitial lung disease, and desquamative interstitial pneumonia: different entities or part of the spectrum of the same disease process?. AJR Am J Roentgenol.

